# Thermal Resonance and Cell Behavior

**DOI:** 10.3390/e22070774

**Published:** 2020-07-16

**Authors:** Umberto Lucia, Giulia Grisolia

**Affiliations:** Dipartimento Energia “Galileo Ferraris”, Politecnico di Torino, Corso Duca degli Abruzzi 24, 10129 Torino, Italy; giulia.grisolia@polito.it

**Keywords:** biothermodynamics, complex systems, thermodynamics of biological systems, biophysical resonance

## Abstract

From a thermodynamic point of view, living cell life is no more than a cyclic process. It starts with the newly separated daughter cells and restarts when the next generations grow as free entities. During this cycle, the cell changes its entropy. In cancer, the growth control is damaged. In this paper, we analyze the role of the volume–area ratio in the cell in relation to the heat exchange between cell and its environment in order to point out its effect on cancer growth. The result holds to a possible control of the cancer growth based on the heat exchanged by the cancer toward its environment and the membrane potential variation, with the consequence of controlling the ions fluxes and the related biochemical reactions. This second law approach could represent a starting point for a possible future support for the anticancer therapies, in order to improve their effectiveness for the untreatable cancers.

## 1. Introduction

The environment in which living cells develop their life slowly changes its chemical and physical properties. In mammalian animals, organs and tissues are generally endowed with a homeostatic capability, which is characterized by internal thermal regulation. Information content has been recently related to the energy flux with the conclusion that, in biology, for all life forms, the message of the genotype encoded in DNA specifies the phenotype. Moreover, it has also been pointed out that information transmission has thermodynamic components associated with data storage and transmission without any additional thermodynamic cost [1,2]. Network properties, such as network entropy and unbalanced motifs, associated with tumor initiation, progression, and anticancer drug responses, have been studied that highlight new potential network-based prognostic and predictive measure in cancer [3,4,5,6,7,8].

A living system can be defined as an open system, so non-equilibrium thermodynamics can be applied to this kind of system [9].

A cancer cell is a living cell that presents a different growth behavior from that of a healthy cell. At a definite phase of its life, any cell (cancer cells included) divides into two daughter cells, which restart their cycle of life. This gives rise to a cyclic process. The single cell size at the beginning of division can vary [10]; consequently, also the relative size of the two daughter cells can vary, but lower and upper bounds on cell volume have been observed [11].

Thus, a good tool for the analysis of cancer can be the thermodynamics of cycle processes. Moreover, in thermodynamics, the definition and the choice of the control volume play a fundamental role. So, in this context, it could be useful to consider the single cell as the observed control volume. Unfortunately, the usual experimental setup does not allow us to introduce such an approach, so we are forced to observe a multitude of single cells and consider this multitude as a complex cooperative system constituted by single cells. This system, considered as a single cell or as the cooperative system of all cells in a culture, from a thermodynamic standpoint represents an open system. Indeed, energy and matter flow through the bound of the system in order to maintain living conditions, while biochemical and biophysical transformations occur within the system, with a related net production of entropy. As regards the environment of the system considered, it is composed by the suspending aqueous solution of cell nutrients, the substances discarded by the cells, and the gaseous atmosphere above the suspending solution. Consequently, the biosystem composition can be defined as:

- The cell membrane, which delimits the volume of the cell, controlling the inflows and outflows of molecules;
- The cytoplasm, which is an aqueous solution of molecules that fills the cell interior;
- The organelles suspended in the cytoplasm.

The system may contain many substances not initially present in its environment. Many enzymes found in fragments of cytoplasm membranes are often not directly accessible to the system environment, while in the environment, the concentrations of some molecular species decrease in time, and nutrients must flow into the system in order to allow the occurrence of biochemical reactions and the production of macromolecular cell components, which lead to an increase of the mass and volume of the living cell [12]. In order to occur, these reactions require an amount of energy that derives from the biochemical reactions themselves, involving nutrients and a related waste of heat toward the living cell environment. The net effect of all these biochemical reactions is the reduction of the entropy of the system, which means increasing the entropy generation in the environment [13].

The analysis of the chemical species and their reactions in the living cells have led to some sequences that start with nutrient molecules and end with the formation of living cells substance and waste molecules and waste heat. Too numerous reaction sequences are considered to exist. Moreover, many molecules can be part of more than one reaction’s sequence, providing a coupling of sequences.

The characteristics of the living systems have been determined by using batch cultures, grown at the optimum temperature for the species employed by the experimenter. This experimental method presents the disadvantages caused by forcing the living system to live in an environment subject to continuous changes, due to the supply and decrease of the environmental concentration of nutrients related to the cell’s growth, while both the environmental concentration of the waste molecules and the waste heat rise proportionally.

The control of fluxes through the cell membrane is achieved by means of endogenous electric fields; in this way, ions and molecules can be accumulated in an nm-thin layer of water.

In 1931, cancer cells were proved to be fermentative, as a consequence of a metabolic injury [14] and genetic effects. In normal cells, mitosis is synchronized with cell growth in order to maintain their size during replication. This biomedical result represents the bioenergetic bases to link the production of lactic acid and extracellular–intratumoral acidification to cancer growth and metastasis. Moreover, both the pH of the cell cytoplasm and the extracellular environment are controlled by the living cell membrane potential.

Differentiated cells are hyperpolarized as compared to quiescent or cycling cells, and the hyperpolarization increases the efflux of some ions (Ca^2+^, K^+^, Zn^2+^, etc.). The fundamental role of inhibition of proliferation in normal as well as neoplastic cells has recently been pointed out as well as the interactions between the ion channels and the other elements of the signaling network. Direct cell migration is fundamental in tissue formation, but if proliferation and invasion is out of control, then a new behavior occurs, and cancer emerges as a disease of abnormal growth. All these processes are driven by fluxes of energy and mass, and the cell shape results are fundamental in their analysis. So, as previously mentioned, from a thermodynamic point of view, living systems are no more than complex systems that are open with a fluxes control.

Considering the chemical reaction at constant pressure and temperature, the Gibbs free energy could seem to be the selected function for the study of the steady states of the living systems. However, its decrease as a criterion for the occurrence of a spontaneous evolution is limited to the complex phenomena that occur at constant temperature and pressure inside the living system. So, a general objective function for the analysis of the living systems is required. Moreover, considering that the system wastes energy, and mass, it generates irreversibility, so the general criterion for the study of its spontaneous evolution is the entropy generation related to the changes of the system. Entropy generation always increases in any spontaneous and irreversible evolution.

The aim of this paper is to deeply analyze the use of the heat role in the study of the cancer systems. Indeed, recently, we have used the entropy generation to introduce a thermodynamic approach to the analysis of the cancer system in order to design a possible support to the present anticancer therapies. In this paper, the authors explain and improve the thermodynamic formulation of this approach, with the second aim of highlighting how to support the biomedical sciences in the comprehension of the possible thermodynamic support to the present anticancer therapies.

## 2. Materials and Methods

Life involves organizational and thermodynamic processes, which tend toward the maximum conversion of available energy [15,16]. The biochemical reactions produce or consume external metabolite, and connect internal metabolites at constant concentrations in the cells at their steady states. Consequently, cells must exchange energy and matter through their membranes [17], which are required for many processes such as replication, transcription, and translation. All these fluxes are driven by the endogenous electric fields, accumulate in the nm-thin layer of water [18,19], and induce biochemical reactions within cells and tissues.

Moreover, the 1931 Nobel laureate Otto Warburg proved that cancer cells are fermentative, pointing out that this was the consequence of a metabolic injury [20]. Even if the tumor behavior is more complex and it is probably based also on genetic structures of the cells, the results of Warburg highlight the important role of energy conversion in cells. This result represents the starting point of our thermodynamic analysis. Indeed, the genetic processes have the consequence of regulation for the cell behavior, but if we consider the cell as a black box, as usually done in thermodynamics, then the genetic regulation can be considered as the “mind” of the cell, without any direct consequence on the thermodynamic balances. This approach allows us to evaluate the life cycle of the cell by considering only the energy and mass fluxes balances during the whole cycle of cell life, and not considering the gene activities, but evaluating only their consequences expressed by the energy conversion in cell.

What are the consequences of fluxes of matter and heat involved in metabolic reactions? To answer this question, we consider the Gibbs free energy variation in time, due to the mass fluxes. The result is [18,21,22,23]:

(1) $$\frac{dG}{dt}=\dot{W}+\sum_{in}{\dot{n}}_{in}{\tilde{\mu }}_{in}-\sum_{out}{\dot{n}}_{out}{\tilde{\mu }}_{out}+T_{0}{\dot{S}}_{g}$$

where G is the Gibbs free energy, $\dot{W}$ is the useful power done by the cell, $\dot{n}$ is the molar flow, $\tilde{\mu }=\mu +Ze\phi$ is the molar electrochemical potential, with μ chemical potential, Ze is the charge of the ion considered, ϕ is the cell membrane electric potential, $T_{0}$ is the temperature of the cell environment, t is the time, in means inflow, out means outflow, and ${\dot{S}}_{g}$ is the entropy generation rate, which is defined as [18,21,22,23]:

(2) $${\dot{S}}_{g}=\frac{dS}{dt}-\frac{\dot{Q}}{T_{0}}-\sum_{in}{\dot{n}}_{in}{\bar{s}}_{in}+\sum_{out}{\dot{n}}_{out}{\bar{s}}_{out}+\sum_{f}{\dot{n}}_{f}{\bar{s}}_{f}$$

where *S* is entropy, $\dot{Q}$ is the heat power exchanged, $\bar{s}$ is the molar specific entropy, and the subscript f means formed. This equation highlights as these flows cause entropy variation; moreover, these fluxes imply also a great number of chemical reactions within the cells, which is accompanied by entropy generation. Now, introducing Equation (2) into Equation (1), we obtain:

(3) $$\frac{dG}{dt}=\dot{W}-\dot{Q}+\sum_{in}{\dot{n}}_{in}\left({\tilde{\mu }}_{in}-T_{0}{\bar{s}}_{in}\right)-\sum_{out}{\dot{n}}_{out}\left({\tilde{\mu }}_{out}-T_{0}{\bar{s}}_{out}\right)+\sum_{f}{\dot{n}}_{f}{\bar{s}}_{f}+T_{0}\frac{dS}{dt}$$

Cell life can be considered as a thermodynamic stationary state, at constant environmental temperature and pressure, and this can be obtained by introducing the following two conditions [18,21,22,23]:

(4) $$\left\{ \begin{array}{l} \frac{dG}{dt}=0\\ \frac{dS}{dt}=0 \end{array}\right.$$

which hold to the equation:

(5) $$\dot{Q}=\dot{W}+\sum_{in}{\dot{n}}_{in}\left({\tilde{\mu }}_{in}-T_{0}{\bar{s}}_{in}\right)-\sum_{out}{\dot{n}}_{out}\left({\tilde{\mu }}_{out}-T_{0}{\bar{s}}_{out}\right)+\sum_{f}{\dot{n}}_{f}{\bar{s}}_{f}$$

which expresses the fundamental role of heat fluxes between the cell and its environment.

The numerical evaluation of Equation (5) is difficult to perform. Indeed, it implies the knowledge of all the balance at the second member of the equation for each cell. So, it is necessary to find an alternative way to evaluate the cell heat flux.

To do so, we consider that living cells’ metabolism implies flows of matter and heat into and out of the cells; the heat flux is the heat wasted by the cell toward its environment. The general approach to the heat transfer is the lumped element model to black box, which holds to the following equation [17,24]:

(6) $$\left\{ \begin{array}{l} \frac{\partial^{2}T_{cell}}{\partial r^{2}}-\frac{H_{M}}{\lambda }=\frac{1}{a}\frac{\partial T_{cell}}{\partial t}\\ \frac{\partial T_{cell}}{\partial t}=-\frac{T_{cell}-T_{0}}{\tau } \end{array}\right.$$

where *r* is a radial variable, considering the cell as a theoretical sphere, *T_cell_* is the temperature, *H_M_* is the metabolism, *a* = *λ*/*ρc_cell_*, in which *ρ* is the density and *c_cell_* is the specific heat, *T*_0_ is the environmental temperature, *τ* = *ρc_cell_ V*/*αA*, *V* is the volume and *A* is the area of the cell, and *α* is the coefficient of convection. This equation leads to a harmonic solution [17,24]:

(7) $$T_{cell}\left(r\right)=T_{0}+\left(T_{cell}-T_{0}\right)\sin\left(\frac{r}{\sqrt{a\tau }}\right)-\frac{a\tau H_{M}}{\lambda }$$

*H_M_* can also include the mitochondrial metabolic activity producing heat. Some relevant data of the cells are calculated [15] as the mass density *ρ* ≈ 10^3^ kg m^−3^, the specific heat *c* ≈ 0.6 J kg^−1^K^−1^, α=0.023Re0.8Pr0.35λ/〈R〉 where *λ* ≈ 0.6 W m^−1^K^−1^ is the conductivity, Re ≈ 0.2 is the Reynolds’ number, Pr ≈ 0.7 is Prandt’s number. In relation to these data, we can evaluate the Biot number Bi ≈ 0.0056; therefore, we can study the heat transfer by using the lumped element model.

As mentioned above, the heat flux represents the heat power exchanged by the cell and its environment. Considering the experimental setup usually used in the biophysical and biochemical analysis of cells, the heat flux is exchanged by convection with the suspending aqueous solution around any cell, so it is possible to write [17,24]:

(8) $$\dot{Q}=\rho_{cell}Vc_{cell}\frac{dT_{cell}}{dt}=\alpha A\left(T_{cell}-T_{0}\right)=\alpha \frac{V}{\langle R\rangle }\left(T_{cell}-T_{0}\right)$$

where $\rho_{cell}$ is the cell mass density, V is the volume of the cell, $c_{cell}$ is the specific heat of the cell, *T_cell_* is the cell’s temperature, α is the coefficient of convection, A=V/〈R〉 is the surface area of the cell, which changes during the phases of the development of the cell, 〈R〉 is the volume/area ratio, a parameter which influences the chemical reaction’s time and the fluxes through the cell membrane, and $\left(T_{cell}-T_{0}\right)$ is the difference of temperatures between the cell temperature and the environment temperature (*T*_0_). The term A=V/〈R〉 is the geometric shape of the cell in relation to convection. We must introduce this quantity because at a stage of its life a cell has a definite volume, but it can change its shape in relation to its duplication phase at that time. Indeed, in eukaryotic cells, the main control process occurs at the G1/S transition, in late S (DNA synthesis) phase, at mitosis (M) entry, and at the metaphase to anaphase transition. Any process is controlled by the cyclin-dependent kinases, which are regulated by the oscillatory expression of G1 and G1/S-cyclins, S-cyclins, and M-cyclins. The transition between metaphase to anaphase is triggered by the anaphase-promoting complex/cyclosome (APC/C). Mitogens stimulate the entry into the cell cycle from a quiescent (G0) phase. Exit from mitosis can lead to differentiation, apoptosis, or return to quiescence [25]. These mechanisms are altered in neoplastic cells.

Now, from Equation (8), we obtain [17]:

(9) $$\frac{d\ln\left(T_{cell}-T_{0}\right)}{dt}=\frac{\alpha }{\rho_{cell}c_{cell}}\frac{1}{\langle R\rangle }$$

with the result that the greater the volume–area ratio, the lower the thermal exchange, when α, $\rho_{cell}$, and $c_{cell}$ are approximately constant. It occurs because the cell adapts its volume/area rate in order to optimize the cell membrane fluxes to obtain the work needed, and conversely, this geometric rate controls also the heat exchange.

## 3. Results

The results obtained highlight the role of the volume–area ratio of the cells in relation to their heath exchange in in vitro experiments. This effect is fundamental when the heat exchange plays a crucial role in the analysis of the experimental data.

In particular, we wish to highlight that this effect is particularly interesting in the study of the cancer growth control compared with normal cell growth, because cancer presents a different metabolic cycle. This is important in the study of cancer growth control. Indeed, we can point out that Equation (9) is also the equation that links a frequency $\nu =1/\tau =\left(\alpha /\rho_{cell}c_{cell}\langle R\rangle \right)$ [17]; i.e., the inverse of the characteristic time to the structural and geometrical properties of the cell and its environment, in relation to the heat exchange. However, what is this frequency?

It is difficult to find an answer without considering thermodynamics. Indeed, each system presents a proper time of response to the external thermal perturbation. We suggest that this frequency is the inverse of the cell proper time of answering to the external thermal perturbation, or the heat exchange rate. Indeed, the heat flow can be also written as:

(10) $$\dot{Q}=\frac{Q}{\tau }=Q\nu$$

where Q is the heat wasted by the cell. Moreover, we can consider also that the fluxes of ions are controlled by the cell membrane potential, which is in turn related to the Gibbs free energy by the following relation [21,22,23]:

(11) $$dG=d\phi -2.3\frac{RT_{0}}{F}d\mathrm{pH}$$

where *ϕ* is the cell membrane electric potential, *R* is the universal constant of gasses, *F* is the Faraday constant, and pH is the potential of hydrogen. At the stationary states, remembering relation (4), it follows:

(12) $$d\phi =2.3\frac{RT_{0}}{F}d\mathrm{pH}$$

which links the variation of the cell membrane electric potential to the variation of the pH, which itself is related to the ion fluxes. Conversely, we can try to force a variation of the pH by a variation of the cell membrane electric potential. Indeed, the concentration of a chemical species follows the law [24]:

(13) $$c_{out}=c_{in}\exp\left(\frac{\Delta \phi }{RT}\right)$$

where *c_out_* and *c_in_* are the concentrations of any ion species outside and inside the cell membrane; *ϕ* is the electric field between the two sides of the membrane, *R* = 8314 J mol^−1^K^−1^ is the universal constant of gasses, *T* is the temperature, and the concentration is related to the pH variation in any cell. In this context, now, we can show as an example the ATP synthase, considering the biochemical reaction:

(14) $$\begin{array}{l} \mathrm{ATP}+H_{2}O\to \mathrm{ADP}+P\\ H_{\mathrm{out}}^{+}\to H_{\mathrm{in}}^{+} \end{array}$$

This biochemical reaction modulates pH by the inflow of H^+^ ions, with the related concentration variation on the border of the cell. Consequently, the membrane potential changes following Relation (11). So, cell functions are known to be regulated by membrane proteins, which are molecules sensitive to the electric field. As a consequence, any change in the membrane electric field, related to the membrane potential, can be transduced into a conformational change of the biological molecules. So, we can argue that this allosteric effect is able to trigger the function of membrane proteins themselves and the relative regulation of cell functions or even entire phenotypes.

**Figure 1:**
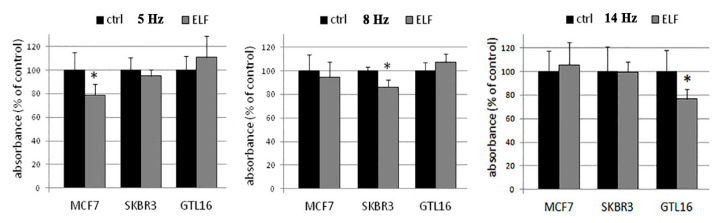
Cell growth and ELF-EMF frequencies.

The Figure 1 highlights the behavior of the cells in relation to the growth and the ELF frequencies [26].

How can we try to do so? We can induce a variation in the cell membrane electric potential by using an electromagnetic wave, with a frequency just equal to the proper frequency of our system.

The results are summarized in Table 1, where three different tumoral cellular lines have been considered [26]: two human breast cancer cell lines both growing in epithelial-like clusters (MCF7 and SKBR3) and one human gastric cancer cell line (GTL16). The two breast cancer lines’ results different in their oncogene expression and estrogen stimulation, and they present different geometry, shapes, and characteristics; in particular, MCF7 is the most commonly used breast cell line [27], especially as concerns the studies of hormone response [28]. GTL16 is a gastric carcinoma cell line with a morphology that is typically round shaped. This cell line presents a rapid growth and the overexpression of the MET oncogene [29,30]. It is possible to highlight that the electromagnetic waves induce a different behavior in the cancer cells considered; indeed, they decrease their growth if compared with the cancer cells outside of the electromagnetic field as shown in Figure 1, proving that there exists a forcing phenomena of heat flux control that controls the related ions fluxes and, consequently, induces a different behavior to the biosystem.

## 4. Discussion

The results here obtained point out the fundamental role of the cell volume–area ratio in relation to the fluxes control.

Indeed, there is a temperature difference between the interior of a living cell and its environment. This is a thermodynamic necessity for life. Sensible heat is exchanged between the inside and outside of the cell due to this temperature difference. This heat flow contributes to entropy generation. Part of the entropy generated appears outside the cell as sensible heat. The fraction of all entropy generation that appears in this form depends on the nature and number of processes occurring within the cell. The consideration of the temperature difference between the environment and cell interior allows the introduction of non-equilibrium thermodynamics for the analysis of cells’ behavior. Brock suggested that the stability of thermophilic organisms can be attributed to the membrane structure properties of these organisms [31].

The gradient of temperature contributes to the flow of substances through the cell membranes of the cell with a consequent influence on metabolic processes [32,33,34]. The approach here suggested allows us to evaluate the homeostatic cellular response to external perturbations. This answer is a thermochemical output of the cell toward the environment. So, we can suggest that the thermodynamic approach holds to a model of analysis of the action and reaction in terms of membrane flux variations. This approach could represent a new approach to design a possible support to the present anticancer therapies by introducing external fields variation at the proper answer time in the therapeutic protocols. From the experimental results, there is a clear reduction of the growth of the cancer with the consequence of improving the effects of the present therapies.

The growth rate of cells, cancer included, at a fixed temperature is a function of both composition of the medium and chemical potentials of the component substances. This represents a sort of control to the growth, because there is a maximum rate at which each biochemical reaction can occur under the existing constraints. This rate is conditioned by the volume–area ratio because it controls the ions fluxes—i.e., the fluxes of the chemical reactants.

So, our results show a method for the design of therapies and experiments for their analysis. Indeed, the specific effect of the single frequencies has two important consequences:

- In each cell type, different parameters of electromagnetic waves impact diversely on the energy utilization and cell proliferation, with different inhibition effects on the cell growth;
- The same electromagnetic wave has distinct effects on different cells, with a selectivity behavior.

Recently, the Bandyopadhyay research team has demonstrated that the spontaneous oscillations of neurons microtubules of the frequency of around 1 MHz oscillation of electrical dipole moments of free electrons and conformational switching cause wave interference, which is the origin of the characteristic shape of the electrical oscillations of the brain at the electroencephalographic signal of 4–40 Hz nested gestalts, which are named beat frequencies [34,35]. The result is no more than the link between the brain synchronization of consciousness with the quantum mechanical behaviors of microtubules. This outcome proves that quantum vibrations microtubules are entangled across neuronal networks via the gap junction, interconnecting channels that physically link neurons together. This result highlights the fundamental role of microtubules, and their quantum effect, with particular interest for resonance, in cell behavior. However, the proven theory does not suggest any link between the microscopic and macroscopic behaviors of cells. This can be considered a thermodynamic resonance for the whole cell. Microtubules are electrical polar structures with power supply from hydrolysis (around 10–14 W cm^−1^ per unit length of the microtubule) of guanosine triphosphate to guanosine diphosphate: the related energy can excite vibrations. Microtubules lose part of the energy by viscous damping of the surrounding cytosol [36]. Microtubules have a crucial role both in the organization activities of the living cell’s cytoskeleton and in the intracellular transport [37]. Bandyopadhyay showed microtubules with a frequency of around 1 MHz, but the global effect of the neurons–microtubules synchronization is the brain 4–40 Hz signal, which is more than five orders of magnitude lower. In particular, Poznanski et al. pointed out how the intracellular capacitive effects of bound electrical charges within mitochondrial membranes can influence electrotonic signals expressed as solitary waves [38], and that the outer mitochondrial membrane acts as an amplifier of the ingoing waves. Moreover, they showed the fundamental role of the changes of the mitochondrial membrane equilibrium potential in sustaining solitons with self-regulation in their amplitude [26]. In physics and chemistry, resonance is the phenomenon in which a vibrating system or external force drives another system to oscillate with a greater amplitude at specific frequencies. At resonant frequencies, small periodic driving forces have the ability to produce large-amplitude oscillations, due to the storage of vibration energy. So, the effect shown by Bandyopadhyay is no more that the amplification of a resonance electromagnetic interaction between external electromagnetic waves and a cell’s microtubules, which generates also the microtubules synchronisation. This is a biological resonant effect!

However, microtubules are in all cells, and they play the same role in all human cells. So, what Bandyopadhyay has pointed out must occur in all cells, with different global effects in relation to their specialized functions. In particular, the mitochondrial respiratory chain and oxidative phosphorylation cause a dispersion of energy as heat. This is related to the energy from nutrients converted in a proton-motive force driving ATP synthesis, which is necessary to transport molecules against the gradient [39]. In this context, the introduction of a thermodynamic approach that realizes this biophysical model can be obtained considering cells as adaptive thermal engines that are able to convert energy from one form to another by coupling metabolic and chemical reactions with transport processes [33]. Human cells must exchange their wasted heat with a constant temperature environment (the human body around them), so if a difference in the metabolism and in the efficiency of the cell system occurs, as in cancer, the cell encounters a difficulty in maintaining its optimal inside temperature for life. However, in any heat exchange, there exists a characteristic temperature function of physical (density, specific heat, convective coefficient) and geometrical (cell volume and membrane area) quantities. This time is a specific time for each cell line. Moreover, this time is also the resonant time, and its inverse is its biological specific frequency. So, by inflowing an electromagnetic wave with a frequency evaluated by the biological specific frequency for any cell line, we can obtain a forced behavior of the considered cell line, which is a modification in the inside organizational process due to the synchronization of the microtubules involved in the resonant interaction, just as in the Bandyopadhyay effect. So, an external short frequency can produce a high microtubules resonant effect that can control the cell’s behavior. The cells reach their optimal asset by a selective process of interactions with their environment, with the consequent effect of the redistribution of energy and mass flows in their metabolic network, enabled by regulatory proteins [33]. Indeed, the cell mitotic cycle is composed of a sequence of processes such as DNA replication, chromosome condensation and segregation, duplication and migration of the spindle pole, breakdown of the nuclear envelope, and cytokinesis [25]. Moreover, the cell cycle has been highlighted as being controlled by a control system that monitors DNA integrity before any transition to the next phase. As regards eukaryotic cells, the main control process occurs at the G1/S transition, in late S (DNA synthesis) phase, at mitosis (M) entry, and at the metaphase-to-anaphase transition. Any process is controlled by the cyclin-dependent kinases, which are regulated by the oscillatory expression of G1 and G1/S-cyclins, S-cyclins, and M-cyclins. The transition between metaphase to anaphase is triggered by the anaphase-promoting complex/cyclosome (APC/C). Mitogens stimulate the entry into the cell cycle from a quiescent (G0) phase. Exit from mitosis can lead to differentiation, apoptosis, or return to quiescence [25]. These mechanisms are altered in neoplastic cells. Cell metabolism is constrained by the maximum amount of macromolecules that can be contained in the intracellular volume. Moreover, it was experimentally pointed out that:

- Any increase in K^+^-channel expression and the activity of K^+^ at the G1/S boundary is often necessary for cell cycles;
- Ca^2+^ fluxes, which can bind with tubulin, control the membrane potential, regulate the mitotic spindle and cytokinesis, regulate DNA transcription, and modulate the expression and activity of the transcription factors that control the expression of the G1 cyclins, producing direct effects on cyclins, cyclin kinases, and the associated proteins;
- Ions fluxes vary the membrane potential, which determines pH variation inside and outside the cell, with a consequent variation of the metabolic cycle [39,40].

## 5. Conclusions

In this paper, we have developed the analysis of a thermodynamic approach to cancer cells, with particular regards to the role of the volume–area ratio in the heat exchange and the consequences to the cancer cells’ behavior.

We have pointed out the existence of a proper time of response of any cell line to the heat exchange. These time results are related to the cell’s volume–area ratio, which is a geometrical parameter that is fundamental for the considerations on the fluxes and cell’s membrane electric potential variation.

Then, starting from some previous experimental results [33], we have obtained also an experimental proof of the present results.

The results highlight how the irreversibility plays a fundamental role also in biophysical systems; indeed, the geometrical rate is completely related to the entropy generation, as it is clear by introducing relation (8) into relation (2). This holds to a new approach to biological physics, based on the first and second law of thermodynamics.

Moreover, the results here obtained confirm the biochemical ones by other scientists who used an entropic approach. Indeed, Luo has demonstrated that a low-frequency and low-intensity electromagnetic field (or ultrasound irradiation) may increase the entropy production rate of a cell in normal tissue than that in cancer. As a consequence, the direction of entropy current between two kinds of cells is reversed. Furthermore, the modification of pH value of cells may also cause the reversal of the direction of entropy flow between healthy and cancerous cells [39,40].

## Figures and Tables

**Table 1:** Cell parameters and calculated ELF-EMF frequencies.

Cell Line	Cell Size	Cell Volume	Volume–Area Ratio	Mean Frequency	Experimental Frequency
	[μm^2^]	[μm^3^]		[Hz]	[Hz]
	1993 ± 16	16,468 ± 793	8.26 ± 0.46		
MCF7	1051 ± 13	17,303 ± 1040	16.45 ± 1.18	5.0 ± 0.7	5 ± 1
	2604 ± 21	42,284 ± 2068	16.24 ± 0.93		
	1033 ± 11	1795 ± 97	1.74 ± 0.11		
SKBR3	2066 ± 17	29,048 ± 1301	14.06 ± 0.74	8.0 ± 2.0	8 ± 1
	2454 ± 20	47,594 ± 2168	20.21 ± 1.05		
	1042 ± 11	1300 ± 80	1.25 ± 0.09		
GTL16	1873 ± 15	2630 ± 140	1.40 ± 0.09	14.0 ± 3.0	14 ± 1
	1059 ± 12	1260 ± 77	1.19 ± 0.09		

For each cell line, 30 cells in different fields were evaluated in their size. Cell volumes were estimated and used to calculate the forcing frequencies of ELF-EMF, using data from our previous experimental paper [26].
